# The mitochondrial master regulator gene PGC1alpha in novel sporadic melanoma cell lines: correlations with BRAF mutational status

**DOI:** 10.1186/1479-5876-12-S1-P9

**Published:** 2014-05-06

**Authors:** Gabriella Guida, Immacolata Maida, Anna Ferretta, Tiziana Cocco, Stefania Guida, Paola Zanna, Rossella Labarile, Letizia Porcelli, Amalia Azzariti, Stefania Tommasi, Anna Albano, Sabino Strippoli, Mari C Turpin Sevilla, Raffaele Filotico, Michele Guida

**Affiliations:** 1 Dept. of Basic Medical Sciences, Neurosciences and Sense Organs, School of Medicine, University of Bari, Italy; 2 Clinical Experimental Oncology Laboratory and Medical Oncology Department, National Cancer Institute, Bari, Italy; 3 Unit of Dermatology and Venereology, University of Bari, and P.O. "A. Perrino", Brindisi, Italy

## Background

Metabolic reprogramming is commonly found in cancer but it is poorly understood in melanoma. Recent works [1,2] provided new insights concerning molecular mechanisms involved in mitochondrial biogenesis of melanoma. This work aims to find possible correlations between pathways involved in the onset and progression of the disease in order to provide supporting information in this field. In particular we studied the behaviour of the mitochondrial master regulator gene PGC1alpha in novel sporadic melanoma cell lines and its relations with BRAF mutational status.

## Materials and methods

We studied new cell lines extracted from sporadic metastatic melanomas (hmel1, M3, Mba72) and primary melanomas (hmel9, hmel11), genotyped for genes involved in melanoma development compared to control melanoma cell lines (HBL, LND1) wt for MC1R and BRAF genes. Hmel1, hmel9 and hmel11 have already been described in Zanna et al., 2011 [3] and Zanna et al., 2013 [4]. We evaluated PGC1α levels and some of its mitochondrial target genes and the mitochondrial respiratory capacity, the amount of ROS, and the lactate level. We related these data to BRAF mutational stauts and analyzed MITF and cAMP levels.

## Results

The HBL and LND1 cell lines, wt for BRAF, highly express PGC1alpha while hmel1, hmel9, hmel11, Mba72, M3, presenting BRAF mutations at the V600 residue, show a downregulation of this gene. MITF expression levels were more abundant in HBL and LND1 cell lines with respect to the other cell lines harbouring BRAF mutations. There is a direct correspondence between PGC1alpha and MITF levels: higher levels of PGC1alpha are associated with an enhanced MITF quantity. The analysis of cAMP levels in our melanoma cell lines showed a similar trend, being higher in wt BRAF cell lines compared to the other cell lines.

## Conclusions

Our data confirm the key role of BRAF mutations, MITF and cAMP levels in melanoma biology, suggesting a very important association with the transcriptional co-activator PGC1alpha, involved in energy metabolism and in mitochondrial biogenesis but also in various physiological stimuli that are reprogrammed in melanoma cells. These data support the divergent pathways hypothesis for melanoma, which may require a reappraisal of targeted cancer prevention and target therapeutic activities.
